# In Vitro Evaluation of Endothelial Progenitor Cells from Adipose Tissue as Potential Angiogenic Cell Sources for Bladder Angiogenesis

**DOI:** 10.1371/journal.pone.0117644

**Published:** 2015-02-23

**Authors:** Liuhua Zhou, Jiadong Xia, Xuefeng Qiu, Pengji Wang, Ruipeng Jia, Yun Chen, Bin Yang, Yutian Dai

**Affiliations:** 1 Department of Urology and Andrology, Affiliated Drum Tower Hospital, Nanjing University School of Medicine, Nanjing, Jiangsu, China; 2 Department of Urology, Nanjing First Hospital, Nanjing Medical University, Nanjing, Jiangsu, China; 3 Department of Urology, The First Affiliated Hospital of Nanjing Medical University, Nanjing, Jiangsu, China; 4 Department of Urology, Shanghai Tenth People’s Hospital, Tongji University School of Medicine, Shanghai, China; University of Torino, ITALY

## Abstract

Autologous endothelial progenitor cells (EPCs) might be alternative angiogenic cell sources for vascularization of tissue-engineered bladder, while isolation and culture of EPCs from peripheral blood in adult are usually time-consuming and highly inefficient. Recent evidence has shown that EPCs also exist in the adipose tissue. As adipose tissue is plentiful in the human body and can be easily harvested through a minimally invasive method, the aim of this study was to culture and characterize EPCs from adipose tissue (ADEPCs) and investigate their potential for the neovascularization of tissue-engineered bladder. Adipose stromal vascular fraction (SVF) was isolated and used for the culture of ADEPCs and adipose derived stem cells (ADSCs). After SVF was cultured for one week, ADEPCs with typical cobblestone morphology emerged and could be isolated from ADSCs according to their different responses to trypsinization. Rat bladder smooth muscle cells (RBSMCs) were isolated and cultured from rat bladder. RBSMCs exhibited typical spindle-shaped morphology. ADEPCs had higher proliferative potential than ADSCs and RBSMCs. ADEPCs stained positive for CD34, Stro-1, VEGFR-2, eNOS and CD31 but negative for α-SMA, CD14 and CD45. ADSCs stained positive for CD34, Stro-1 and α-SMA but negative for VEGFR-2, eNOS, CD31, CD14 and CD45. RBSMCs stained only positive for α-SMA. ADEPCs could be expanded from a single cell at an early passage to a cell cluster containing more than 10,000 cells. ADEPCs were able to uptake DiI-Ac-LDL, bind UEA-1 and form capillary-like structures in three-dimensional scaffolds (Matrigel and bladder acellular matrix). ADEPCs were also able to enhance the human umbilical vein endothelial cells’ capability of capillary-like tube formation on Matrigel. Additionally, significantly higher levels of mRNA and protein of vascular endothelial growth factor were found in ADEPCs than in RBSMCs. These results suggest the potential use of ADEPCs as angiogenic cell sources for engineering bladder tissue.

## Introduction

Many patients suffering from congenital and acquired diseases such as exstrophy, trauma, inflammation and cancer, often end up with impairment of bladder structure and function, and eventually are in need of bladder reconstruction. Development of tissue engineering in the past few decades has brought urologists a novel strategy to create new tissues for augmenting the bladder. Even though different degrees of success had been gained in clinical trials, it is just the first step towards the goal of engineering fully structural and functional bladders[1, 2]. Currently, there are still several challenges ahead of us that need to be completely resolved before this technique is widely applied in clinic[3]. Reports have demonstrated that bladder regeneration was unsatisfactory in the central zone of engineered constructs because of the insufficient formation of vascular networks which are capable of delivering oxygen and nutrients[4, 5].

Vascularization of engineered bladder tissue is one of the most urgent challenges in tissue engineering of the bladder. Prevascularization of the engineered construct in vitro using autologous endothelial cells might be a novel approach for the rapid establishment of adequate blood supply after bladder reconstruction[6]. Currently, isolation and culture of endothelial cells usually requires an invasive procedure for vessel harvest, which may lead to donor-site morbidity. A relatively less invasive procedure for obtaining autologous cells is highly desirable[7].

Endothelial progenitor cells (EPCs) were first discovered in the peripheral blood of adults and demonstrated the capability of proliferating, migrating, and differentiating into endothelial lineage cells, as well as the de novo formation of new vessels[8]. The transplantation of EPCs has been widely applied in regenerative medicine for the treatment of ischemic diseases[9]. EPCs also have the potential for being used as cell sources in the vascularization of tissue-engineered bladder. Sharma et al. demonstrated the formation of vasculature in a chorioallantoic membrane model using EPCs[10]. Reports have also showed that EPCs could improve blood supply for bladder regeneration in combination with vascular endothelial growth factor (VEGF) gene therapy[11].

Although autologous EPCs can be isolated and cultured from host blood, the level of EPCs in circulation is very low. It might be time-consuming for cell expansion to obtain a large quantity of source cells for transplantation. Furthermore, it might be impossible to isolate and culture EPCs when diseases with conditions that impair the viability and function of circulating EPCs are present[12]. Recent evidence has shown that EPCs also exist in the adipose tissue[13]. As adipose tissue is plentiful in the human body and can be easily harvested through a minimally invasive method, a large quantity of autologous EPCs can be obtained in a short-term ex vivo expansion[14]. However, to the best of our knowledge, the angiogenic potential of adipose tissue derived EPCs (ADEPCs) in tissue engineering of the bladder has not been evaluated. This current study, therefore, prepared an acellular biological scaffold, isolated and cultured ADEPCs, and then evaluated the angiogenic potential of these cells in three-dimensional (3D) scaffold.

## Materials and Methods

### Animals

All animal procedures were approved by the Institutional Animal Care and Use Committee of the Affiliated Drum Tower Hospital to Nanjing University School of Medicine. The investigation was performed according to the institutional and national guidelines for laboratory animals. A total of 6 male Sprague-Dawley rats (body weight, 250–280 g) were used in this study. Animals were housed in a standard room with a 12 h light/dark cycle and constant humidity and temperature, given food and water *ad libitum*.

### Cell isolation and culture

Stromal vascular fraction (SVF) was isolated from epididymal adipose tissue as previously described but with a modification[15]. Briefly, rats were anesthetized by intraperitoneal injection of pentobarbital (50 mg/kg). After the rats were shaved, cleaned and sterilized, the epididymal adipose tissue was exposed and removed through a midline lower abdominal incision. Then the abdominal wall and skin were closed and the rats were raised and housed as previously described. Retrieved epididymal adipose tissue was rinsed with ice-cold phosphate-buffered saline (PBS, Sigma-Aldrich) containing 10% penicillin and streptomycin for thrice, minced into small pieces, and digested with 0.075% type I collagenase (Sigma-Aldrich) at 37℃ for 40 min with a vigorous shake. After filtration through 200 μm nylon mesh (BD Biosciences) and centrifugation at 400g for 5 min, the cell pellet was treated with Red Blood Cell Lysis Buffer for 1 min, neutralized with ice-cold PBS, and centrifuged again. The cells were collected and resuspended at a density of 2×10^5^/ml, in Dulbecco’s Modified Eagle Medium (DMEM, Gibco), which was supplemented with 10% fetal bovine serum (FBS, Gibco), 1% penicillin and 1% streptomycin, and then cultured in 25 cm^2^ flask.

When most of the primary cells grew until 80% confluence after culture for about one week, endothelial-like colonies could be found within spindle-like cells. After the cells were washed twice with PBS, dissociation solution containing 0.25%Trypsin/0.038% Ethylene Diamine Tetraacetic Acid (EDTA) (Gibco) was added to the flask and incubated for several seconds, during which the cells were carefully monitored under an inverted microscope. The spindle-like cells could be easily dissociated during trypsinization, while endothelial-like colonies still attached firmly to the surface of the flask. The digestion response was then terminated by the addition of DMEM containing 10% FBS. Accordingly, the cells with different response rates to the dissociation solution were separated. The dissociated spindle-like cells (adipose tissue derived stem cells, ADSCs) were subcultured in a new flask. The endothelial-like colonies (ADEPCs) that still adhered to the surface were kept in the initial flask and allowed to grow until confluence. Both the ADSCs and ADEPCs were cultured in DMEM containing 10% FBS.

Rat bladder smooth muscle cells (RBSMCs) were isolated and cultured from the smooth muscle layer of bladder tissue according to our previous protocol[16]. Briefly, the smooth muscle layer of bladder tissue from the rat, after a partial cystectomy, was incubated in 0.25%Trypsin/0.038%EDTA solution at 37℃ for 20 min, cut into pieces, and then treated with 0.1% type I collagenase (Sigma-Aldrich) at 37℃ for another 20 min. The cells were collected and cultured in DMEM/F12 medium (Gibco) containing 10% FBS.

All the three types of cells were cultured at 37℃ humidified atmosphere with 5% CO_2._ The medium was changed every other day until subconfluence for subculture. All the cells used in this study were at the passage 1–5.

### Cell proliferation

Cell proliferation assay was performed as previously described but with a modification[17]. Cell growth pattern, population doublings (PD), and doubling time (DT) were assayed. To calculate PD and DT, cell numbers and culture time were counted at passage 1 and 5. PD and DT were calculated as follows: PD = ln(N_f_/N_i_)/ln(2); DT = C_t_/PD. (N_f_: Final number of cells that were harvested, N_i_: Initial number of cells that were seeded, C_t_: Culture time that derives from the time interval between cell seeding and harvest). This cell proliferation experiment was replicated six times.

### Immunofluorescence staining for cell phenotype

ADEPCs, ADSCs and RBSMCs were cultured on glass slides in a 24-well plate pre-coated with 0.1% gelatin for facilitating cell adhesion. Immunofluorescent staining with primary membranous (CD31, CD34, Abcam) and cytoplasmic (stromal cell antigen [Stro-1], Millipore; endothelial nitric oxide synthase [eNOS], BD Biosciences; alpha-smooth muscle actin [α-SMA], Sigma-Aldrich) antibodies was done. An IgG-matched isotype served as the internal control for each antibody.

### Flow cytometric analysis

ADEPCs and ADSCs were trypsinized, washed three times with PBS containing 1% BSA, and pelleted by centrifugation at 400 g. Cells were incubated with membranous (CD14, CD31, CD34, CD45 and VEGF Recepter-2 (VEGFR-2)) and cytoplasmic (Stro-1, eNOS, and α-SMA) antibodies at RT for 40 min. A fixation/permeabilization kit (BD Biosciences) was applied for staining intracellular antigens. Cells were then washed twice and incubated at RT for 30 min with Alexa Fluor 488-conjugated goat anti-mouse secondary antibody in the dark. The labeled cells were washed twice, resuspended and finally analyzed with FACSCaliber (BD Biosciences). An isotype-matched IgG was set as the control for each primary antibody. The flow cytometric analysis was repeated six times.

### DiI-Ac-LDL uptake and FITC-UEA-1 binding

ADEPCs were plated on glass slides in a 24-well plate as described previously, and cultured until 70–80% confluence. The cells were incubated with 10 μg/ml DiI-acetylated-low-density lipoprotein (DiI-Ac-LDL, Molecular Probes, USA) in culture medium at 37℃ for 4h. Cells were then fixed with 4% formaldehyde and incubated with 10μg/ml Fluorescein isothiocyanate-conjugated Ulex europaeus lectin (FITC-UEA-1, Vector, USA) at RT for 1h. Finally, the cells were stained with DAPI and images were captured under a fluorescent microscope.

### Single-cell clonogenic assay

A single cell clonogenic assay was performed to evaluate the clonogenic capacity as previously described but with a modification[18, 19]. Early ADEPCs (passage 1 to 2) were serially diluted and plated into a 96-well plate pre-coated with type I collagen. The wells were examined under an inverted microscope and only wells containing a single cell were monitored. The wells with a cell clone containing more than 1000 cells (about one week after culture) were subcultured to a 24-well plate. After two weeks culture, wells were examined for colony growth or cell confluence by visual inspection. Colonies were graded according to size into various categories: 2–50, 51–200, 201–2000, 2001–10000, >10000 cells. We randomly selected 5 confluent wells from five different animals for long-term passage in a T75 culture flask. Cultured cells were used for the following examination.

### Real-time quantitative reverse transcription PCR

Real-time quantitative reverse transcription PCR (RT-PCR) was performed to examine VEGF gene expression in ADEPCs, ADSCs and RBSMCs. Total RNA was extracted by using TRIzol reagent (Invitrogen) and converted into cDNA with Prime Script RT Master Mix (TaKaRa) according to the manufacturer’s protocol. Then, a real-time RT-PCR reaction was completed using the SYBR *Premix Ex Taq* (Tli RnaseH Plus) (TaKaRa). Primer sequences were shown in S1 Materials and Methods. The real time RT-PCR assay was repeated six times.

### Enzyme-linked immunosorbent assay

For quantification of secreted VEGF, ADEPCs, ADSCs and RBSMCs were plated in 6-well plates (three wells for each) at a density of 5×10^5^ cells/well respectively. After attachment overnight, the medium was discarded. Cells were then washed twice and cultured in 1 ml of DMEM without FBS for 24h to prepare the conditioned medium. Levels of VEGF in the conditioned medium were determined by Enzyme-linked immunosorbent assay (ELISA) kit (Neobioscience Technology Company) according to the manufacturer’s instruction. The ELISA experiment was replicated six times.

### Cell labeling

ADEPCs were labeled with the lipophilic fluorochrome chloromethylbenzamido dialkylcarbocyanine (CM-DiI, Molecular Probes) according to the manufacturer’s instruction. Briefly, ADEPCs were incubated with CM-DiI at 37℃ for 5 min (density, 1 μg CM-DiI per million cells), followed by incubation at 4℃ for 15 min with occasional mixing, and finally washed twice for subsequent studies.

### Matrigel-based capillary-like tube formation assay

200 μL growth factor reduced Matrigel (BD Biosciences) was added to a 24-well plate and allowed to solidify at 37℃ for 30 min. ADEPCs labeled with CM-DiI were seeded quintuplicate onto the Matrigel at 6×10^4^ cells/well and incubated for 10h to allow the formation of tubes. Three representative images were recorded from each well using phase-contrast and fluorescence microscopy. This experiment was replicated six times.

To compare the pro-angiogenic effects of ADEPCs, ADSCs and RBSMCs on endothelial cells, Matrigel-based capillary-like tube formation assay was performed using human umbilical vein endothelial cells (HUVECs) according to our previous protocol[20]. ADEPCs, ADSCs and RBSMCs conditioned medium (CM) were prepared as previously described[21]. HUVECs were seeded quintuplicate onto solidified Matrigel at 6×10^4^ cells/well for Matrigel-based capillary-like tube formation assay. Cells were incubated with four different medium: DMEM without FBS (control group), ADEPCs CM, ADSCs CM and RBSMCs CM. This experiment was repeated six times.

### Cell-seeded bladder acellular matrix and immunofluorescence analyses

A porcine bladder acellular matrix (BAM) was prepared as our previous protocol described[5, 22]. After labeling with CM-DiI, ADEPCs were seeded on sterilized BAM at 5×10^6^ cells/cm^2^ and incubated for 14 days. The cell-BAM construct was then embedded with Optimal Cutting Temperature compound (OCT; USA), frozen at -20°C. Frozen tissue sections (6μm) were blocked, incubated with anti-eNOS antibody at 4°C overnight, and stained with second antibody conjugated with Alexa Fluor 488. Finally, the cellular nuclei were detected by DAPI staining.

### Statistical analysis

All the data were expressed as mean ± standard error. Data were compared among the groups by using one-way analysis of variance. The Tukey-Kramer test was used for *post-hoc* comparisons (SPSS, version 16.0). A *P*<0.05 was considered significant.

Further detailed information is presented in the S1 Materials and Methods.

## Results

### Cell morphology and growth characteristics

**Fig 1:**
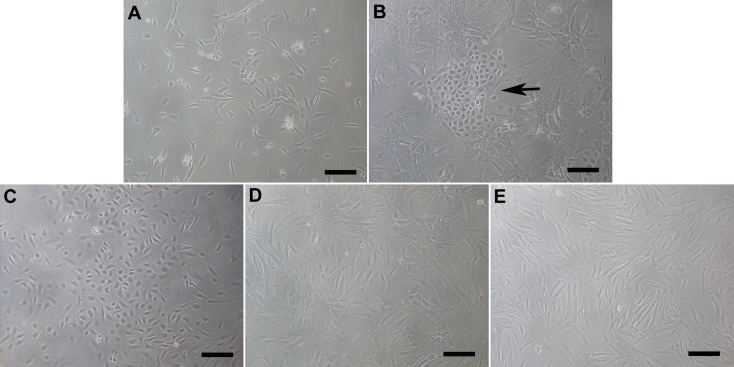
Morphology of ADEPCs, ADSCs and RBSMCs. **(A)** Primary cells appeared after culture for 48h. **(B)** After one-week culture, ADEPCs (black arrow) emerged within spindle-like ADSCs. **(C)** ADEPCs could be separated from ADSCs according to their different responses to trypsinization. **(D)** ADSCs were subcultured in a new flask. **(E)** RBSMCs emerged and exhibited typical spindle-shaped morphology after 5 days culture. Scale bar = 100μm.

After 48h of culture, adherent cells emerged and then proliferated quickly (Fig. 1A). About one week later, endothelial-like colonies with typical cobblestone morphologies (ADEPCs) were present within spindle-like cells (ADSCs) (Fig. 1B). When the ADSCs were dissociated during trypsinization, the endothelial-like ADEPCs colonies still adhered to the flask surface and allowed to grow until confluence (Fig. 1C). The ADSCs were then subcultured in another flask (Fig. 1D). RBSMCs emerged and exhibited typical spindle-shaped morphologies after 5 days culture (Fig. 1E).

**Fig 2:**
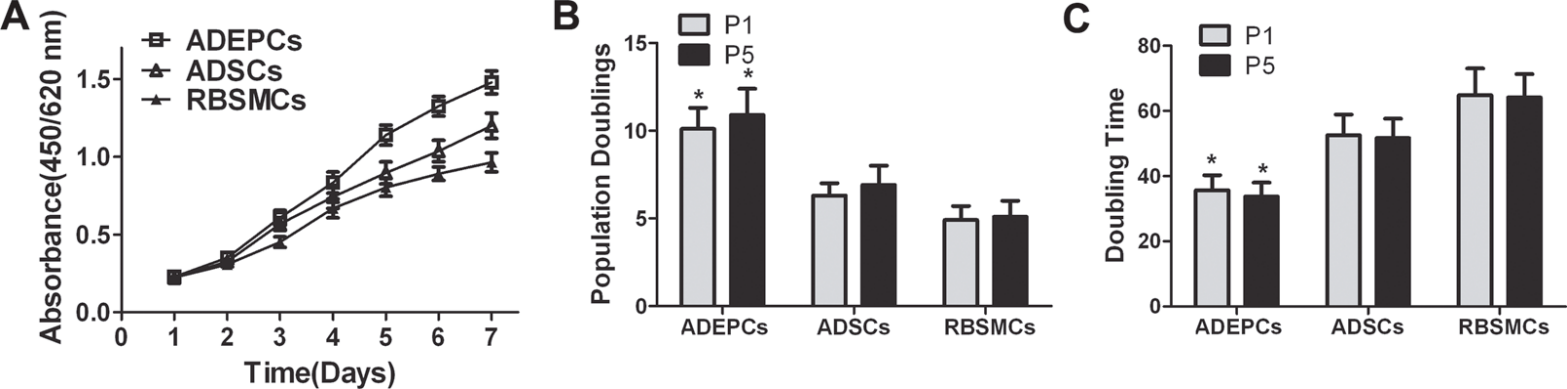
Growth characteristics of ADEPCs, ADSCs and RBSMCs. **(A)** Cell proliferation curve showed that all the three kinds of cells had similar growth pattern. **(B)** The PD at passage 1 and 5 of ADEPCs (10.1±1.2 and 10.9±1.5) were significantly higher than that of ADSCs (6.3±0.7 and 6.9±1.1) and RBSMCs (4.9±0.8 and 5.1±0.9) (*p<0.05, n = 6). **(C)** The DT at passage 1 and 5 of ADEPCs (35.7±4.6 hours and 33.8±4.2 hours) were significantly lower than that of ADSCs (52.6±6.3 hours and 51.7±5.9 hours) and RBSMCs (64.8±8.2 hours and 64.2±7.1 hours) (*p<0.05, n = 6).

All the three kinds of cells had similar growth patterns (Fig. 2A). However, ADEPCs displayed more proliferative potential than ADSCs and RBSMCs. The PD at passage 1 and 5 of ADEPCs were 10.1±1.2 and 10.9±1.5, which were significantly higher than that of ADSCs (6.3±0.7 and 6.9±1.1) and RBSMCs (4.9±0.8 and 5.1±0.9) (p<0.05) (Fig. 2B). The DT at passage 1 and 5 of ADEPCs were 35.7±4.6 hours and 33.8±4.2 hours, which were significantly less than that of ADSCs (52.6±6.3 hours and 51.7±5.9 hours) and RBSMCs (64.8±8.2 hours and 64.2±7.1 hours) (p<0.05) (Fig. 2C).

### Characteristics of ADEPCs

**Fig 3:**
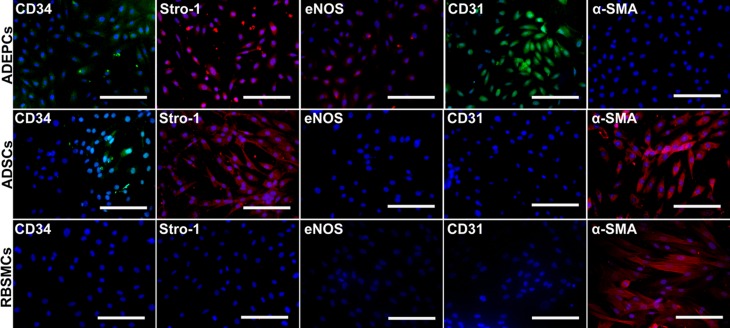
Immunofluorescence staining of ADEPCs, ADSCs and RBSMCs. ADEPCs were stained positively for CD34, Stro-1, eNOS and CD31 but negatively for α-SMA. ADSCs were stained positively for CD34, Stro-1 and α-SMA but negatively for eNOS and CD31. RBSMCs were only stained positively for α-SMA. Scale bar = 100 μm

**Fig 4:**
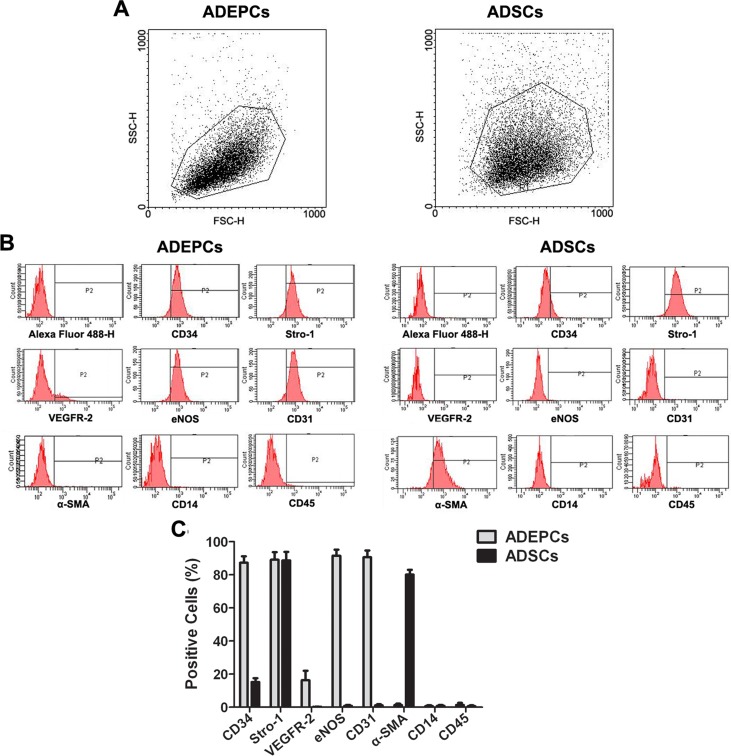
Flow cytometric analysis of ADEPCs and ADSCs. **(A)** The single cell population with high forward scatter of ADEPCs and ADSCs could be obtained through our isolation technique. **(B)** ADEPCs were positive for CD34 (87.3±3.9%), Stro-1 (89.1±4.6%), VEGFR-2 (16.3±5.7%), eNOS (91.5±3.7%) and CD31 (90.6±4.1%) but negative for α-SMA, CD14 and CD45. ADSCs were positive for CD34 (15.2±2.3%), Stro-1 (88.7±5.2%) and α-SMA (80.1±2.9%), but negative for VEGFR-2, eNOS, CD31, CD14 and CD45. **(C)** Quantification of surface markers (means expressed as percentages; n = 6).

Immunofluorescence staining demonstrated that both ADEPCs and ADSCs, but not RBSMCs, expressed CD34 and Stro-1. However, ADEPCs expressed CD31 and eNOS but not α-SMA. ADSCs and RBSMCs expressed α-SMA but not CD31 or eNOS (Fig. 3). These results were further confirmed by flow cytometric analysis of ADEPCs and ADSCs. ADEPCs were positive for CD34 (87.3±3.9%), Stro-1 (89.1±4.6%), VEGFR-2 (16.3±5.7%), eNOS (91.5±3.7%) and CD31 (90.6±4.1%), but negative for α-SMA, CD14 and CD45. ADSCs were positive for CD34 (15.2±2.3%), Stro-1 (88.7±5.2%) and α-SMA (80.1±2.9%), but negative for VEGFR-2, eNOS, CD31, CD14 and CD45. Flow cytometric analysis also showed that the single cell population with high forward scatter of ADEPCs and ADSCs could be obtained through our isolation technique (Fig. 4).

**Fig 5:**
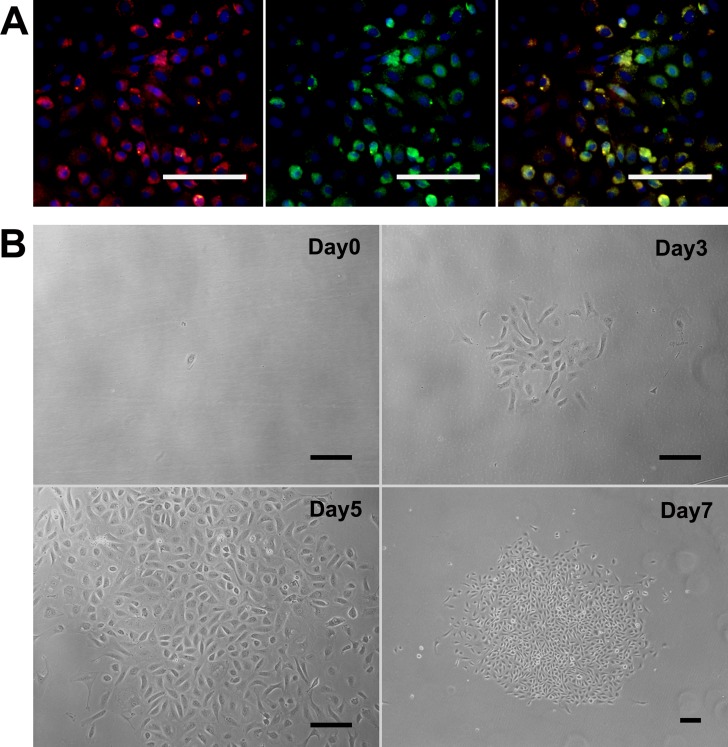
Functional characterization of ADEPCs. **(A)** ADEPCs had the capability for DiI-Ac-LDL uptake (red) and UEA-1 binding (green). Dual positive cells could also be observed (yellow, merged). **(B)** The single-cell clonogenic assay shows that ADEPCs at early passage can expand from one single cell to a new cluster after culture for 3, 5 and 7 days. Scale bar = 100 μm

**Fig 6:**
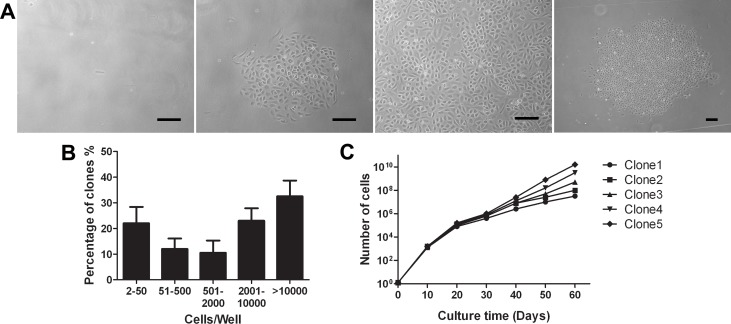
Quantitation of the clonogenic and proliferative potential of single cells derived from ADEPCs colonies. **(A)** Representative photomicrographs of ADEPCs colonies from single cell clones to clusters. **(B)** Number of cell progeny derived from single cells plated on individual wells after 14 days of culture. **(C)** Growth kinetics of the cell progeny of 5 single-plated ADEPCs in long-term culture. Scale bar = 100 μm.

We additionally found that ADEPCs but not ADSCs could incorporate DiI-Ac-LDL and bind UEA-1 (Fig. 5A). In single-cell clonogenic assays, early ADEPCs could expand from a single cell to a cell cluster containing more than 1,000 cells after 7 days culture (Fig. 5B). Photomicrographs of the different size and morphology of the cell clusters derived from a single cell were shown in Fig. 6A. There was a hierarchy of proliferative potential ranging from 2–50 cells to >10000 cells at 2 weeks after single cell seeding. Approximately 30% of the single-plated ADEPCs that divided formed well-circumscribed colonies containing more than 10000 cells (Fig. 6B). We randomly tested the cell progeny of 5 single ADEPCs. Results showed that some ADEPCs could expand from a single cell to about 10^10^ progeny in long-term culture. Meanwhile, all the cells could expand to about 10^7^ cells (Fig. 6C). These results above indicated that ADEPCs have a high potential for proliferation.

### Tube formation of ADEPCs in 3D scaffolds

**Fig 7:**
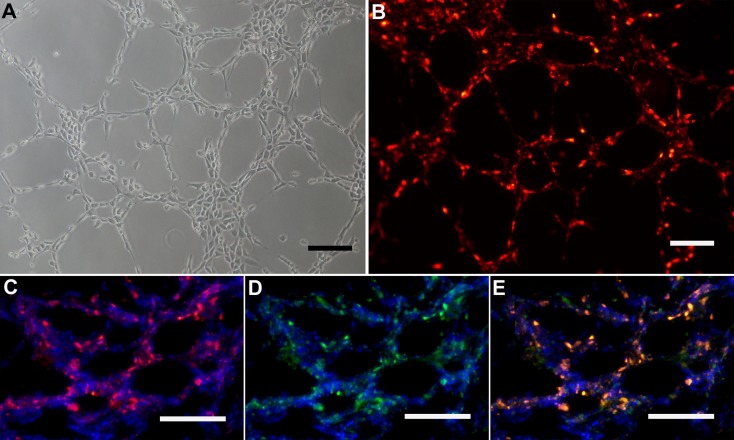
Tube formation of ADEPCs in 3D scaffolds. **(A)** ADEPCs before and after labeling with CM-DiI could form capillary-like structures in the Matrigel. **(B)** After seeding on BAM, ADEPCs labeled with CM-DiI (red) grew on the pore surface within the scaffold, forming capillary-like structures and are still positive for eNOS (green). Dual positive fluorenscence (yellow) for CM-DiI and eNOS could also be observed. The cell nucleus was counterstained with DAPI (blue). Scale bar = 100 μm.

**Fig 8:**
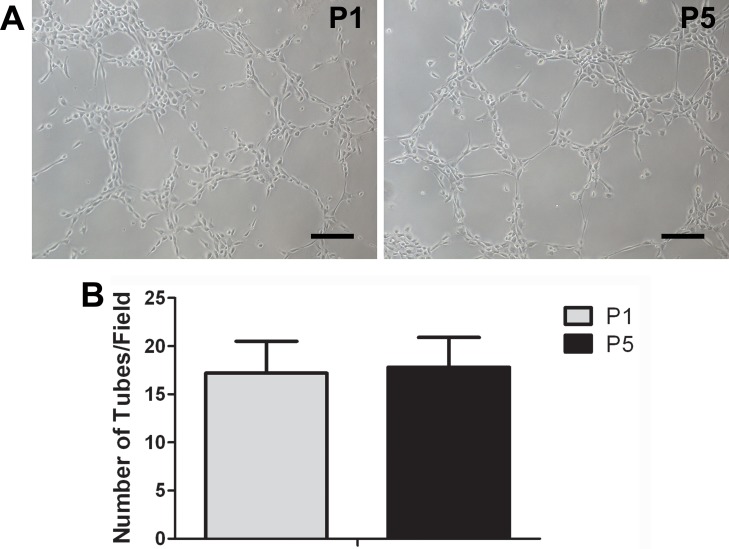
Tube formation apability of ADEPCs at passage 1 and 5. **(A)** Representative photomicrographs of tube formation of ADEPCs at passage 1 (P1) and 5 (P5). Scale bar = 100 μm. **(B)** There was no significant difference on the capability of tube formation of ADEPCs between passage 1 and 5 (p>0.05, n = 6).

Capacity of tube formation in 3D scaffolds (Matrigel and BAM) was evaluated both in labeled and unlabeled ADEPCs. It was found that both labeled and unlabeled ADEPCs could form capillary-like structures in Matrigel (Fig. 7A). After being seeded on BAM, labeled ADEPCs grew on the pore surface within the scaffold, formed capillary-like structures and were still positive for eNOS (Fig. 7B). Additionally, there was no significant difference on the capability of tube formation of ADEPCs between passage 1 and 5 (Number of tubes: 17.2±3.3 vs. 17.8±3.1, p>0.05) (Fig. 8).

### Pro-angiogenic effect of ADEPCs

**Fig 9:**
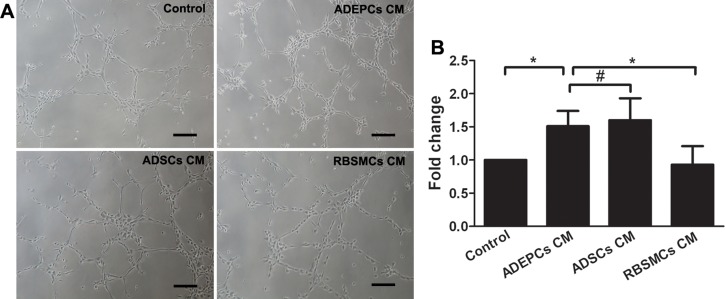
Tube formation of human umbilical vein endothelial cells (HUVECs) treated by the conditioned medium derived from ADEPCs, ADSCs and RBSMCs. **(A)** Representative photomicrographs of tube formation of HUVECs treated by different medium. Scale bar = 100 μm. (B) Quantitative analyses of tube formation of HUVECs among different treatments. The results were expressed as fold change of the control (*p<0.05, #p>0.05, n = 6).

Matrigel-based capillary-like tube formation assay showed that ADEPCs CM were able to significantly enhanced the HUVECs’ capability of capillary-like tube formation than RBSMCs CM (Fold change: 1.51±0.23 for ADEPCs CM vs. 0.93±0.28 for RBSMCs CM, control group served as 1, p<0.05). No significant difference of tube formation was found between ADEPCs CM and ADSCs CM (Fold change: 1.6±0.33 for ADSCs CM, p>0.05) (Fig. 9).

**Fig 10:**
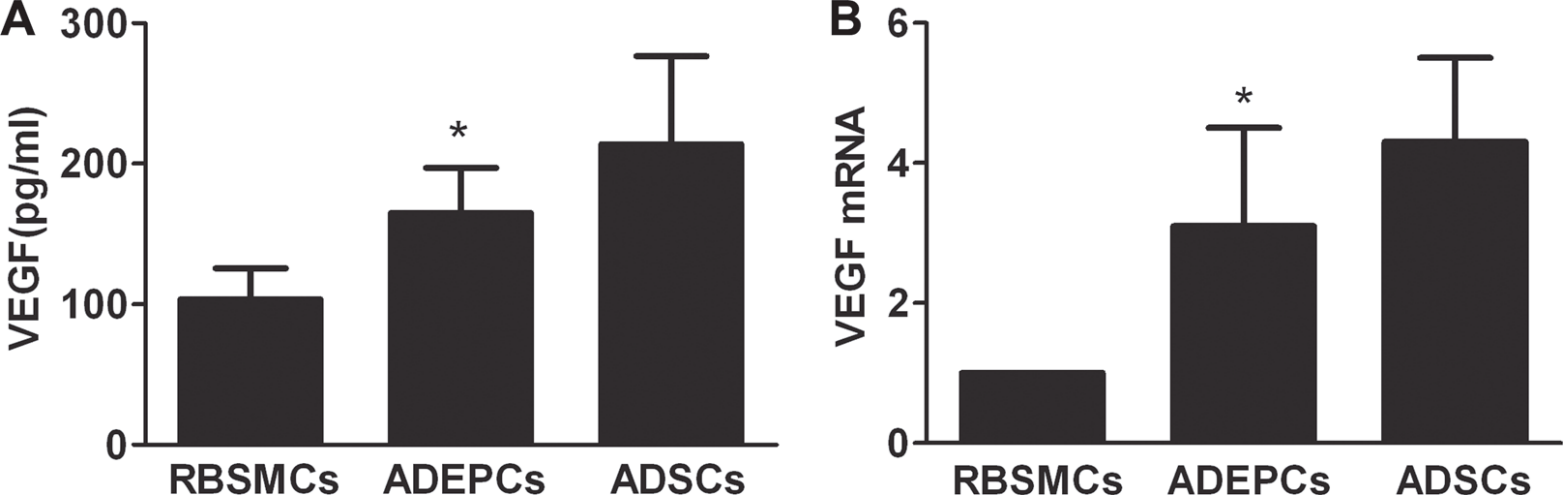
Expression of the mRNA and protein of vascular endothelial growth factor (VEGF) by ADEPCs, ADSCs and RBSMCs. **(A)** ELISA showed that ADEPCs secreted significantly higher VEGF protein than RBSMCs (*p<0.05, vs. RBSMCs, n = 6). However, VEGF secreted from ADEPCs is lower than that from ADSCs (p<0.05, n = 6). **(B)** Real-time RT-PCR indicated that ADEPCs and ADSCs expressed significantly higher VEGF mRNA than RBSMCs (*p<0.05 vs. RBSMCs, n = 6).

ELISA showed that significantly higher VEGF protein was secreted by ADEPCs than RBSMCs (164.8±32.4pg/mL vs. 103.6±21.9pg/mL, p<0.05). However, VEGF secretion from ADEPCs was significantly lower than from ADSCs (213.8±62.7pg/mL, p<0.05). Real-time RT-PCR indicated that ADEPCs and ADSCs expressed significantly higher VEGF mRNA than RBSMCs (3.1±1.4 and 4.3±1.2 times of RBSMCs, respectively, p<0.05) (Fig. 10).

## Discussion

Currently, bladder regeneration is limited by the low revascularization rate of the implanted materials[4]. To enhance the neovascularization of engineered bladder tissue, some angiogenenic growth factors have been used in the rodent bladder augmentation model[23, 24]. However, the revascularization of regenerated bladder was still insufficient when a large bladder area was reconstructed even though two kinds of angiogenic factors were used. Delivery of exogenous growth factor might not be able to rapidly and adequately establish the blood supply for bladder regeneration because the formation of a vascular network depends on the ingrowth of vessels from adjacent native tissue[5]. The transplantation of endothelial cells or their progenitors might be able to prevascularize engineered tissue in vitro and establish vasculature de novo in the regenerated bladder after implantation. It is therefore highly desirable to obtain a source for a large quantity of angiogenic cells through a minimal invasive technology for engineering bladder tissue.

Adipose tissue is plentiful in the human body and has been suggested as an attractive and abundant stem cell source for tissue repair and regeneration[25]. SVF is an important cell population in adipose tissue and contains numerous cell types, including preadipocytes, mature endothelial cells, vascular smooth muscle cells, fibroblasts, plenty of stem/progenitor cells, and so on[26]. Stem cells in adipose tissue have the property of pluripotency and can differentiate into endothelial cells, a process which may be mediated by fibroblast growth factor 2 signaling[27]. Endothelial progenitor cells that serve as a precursor of endothelial cells also exist in SVF. Since the directed differentiation from EPCs to endothelial cells is important for the prevascularization of the engineered construct in vitro, it is necessary to isolate EPCs from adipose SVF.

ADEPCs were first isolated from adipose tissue and cultured by Planat-Benard et al using the differential adhesive ability of EPCs from other cells. After SVF had been plated for six hours, the non-adherent cells could be removed and the endothelial-like cells could arise from the adherent cells[13]. ADEPCs demonstrate a relative resistance to trypsinization so that they can not be easily dissociated during trypsinization. In this study, we had successfully cultured ADEPCs from adipose tissue, and a highly purified population of ADEPCs had been obtained after being seperated from ADSCs based on their different response rates to the dissociation solution.

As the progenitor of endothelial cells, ADEPCs demonstrated the characteristics of both progenitor cells and endothelial cells which were similar to circulating EPCs[28]. Firstly, single ADEPCs at an early passage have a high capacity of self-renewal in single cell clonogenic assay[18]. Secondly, ADEPCs have similar phenotypes to circulating EPCs, and expressed hematopoietic and endothelial markers, such as CD34, Stro-1, VEGFR-2, eNOS and CD31[28]. Stro-1 used to be considered a mesenchymal stem cells marker, however, a recent study reported that it was also an endothelial antigen and expressed in endothelial cells[29]. Interestingly, we also found that Stro-1 is expressed in ADEPCs, which further supports that Stro-1 might intrinsically be an endothelial antigen, and its expression in ADEPCs is probably an induced event[29]. Furthermore, ADEPCs have the capability for DiI-Ac-LDL uptake and UEA-1 binding, which are well known as the properties of EPCs[18, 28].

Theoretically, transplantation of ADEPCs could enhance neovascularization of tissue-engineered bladder through direct and indirect approaches[30]. In this study, we performed tube formation experiments to evaluate, in vitro, whether the ADEPCs have the potential of directly forming new vessels. Our results indicated that ADEPCs formed capillary-like structures in both the Matrigel and acellular biological scaffold, implying that ADEPCs could be used as angiogenic cells for prevascularization in tissue engineering. However, future study should be carried out to investigate whether the newly formed vasculature could integrate into the host vessels after implantation[6]. Cell tracing technologies might be beneficial for such a study[31].

Reports demonstrated that EPCs could secrete angiogenic factors, which could stimulate the proliferation and migration of endothelial cells[32]. In this study, we used Matrigel-based capillary-like tube formation assay to evaluate the pro-angiogenic effects of ADEPCs on endothelial cells, and molecular biological methods to investigate whether ADEPCs could secret VEGF, one of the most important por-angiogenic factors[20]. Results demonstrated that ADEPCs expressed high levels of mRNA and protein of VEGF, implying that transplantation of ADEPCs could have a paracrine effect on host endothelial cells and could indirectly facilitate the formation of new vessels in vivo[9]. However, inconsistent results existed between Matrigel-based capillary-like tube formation and molecular biological assay, which may be attributed to the extensive list of angiogenic factors synthesized by ADEPCs. The synergetic effects of these factors on host endothelial cells need to be further evaluated[16].

Reports also demonstrated that EPCs could produce the extracellular vesicles (EVs), which participate in cell-to-cell communication through transferring proteins, bioactive lipids and nucleic acids, and indirectly contribute to angiogenesis[33]. EPCs derived microvesicles, one of the EVs, were able to trigger an angiogenic program in quiescent endothelial cells by a horizontal transfer of mRNA[34]. In addition, some growth factors may trigger the release of EVs from adipose mesenchymal stem cells and enhance their angiogenic potential[35]. However the role of EVs derived from ADEPCs needs to be further studied.

One limitation of this study is the static culture condition of ADEPCs in Matrigel and BAM. As the dynamic culture condition was better than the static condition in nutrition supply and cell metabolism, future study is needed to investigate the effect of dynamic culture of ADEPCs in BAM on the secretion of angiogenic factors[36]. The second limitation is that only in vitro evaluations were performed. To further investigate the exact roles of ADEPCs in neovascularization of tissue-engineered bladder, an animal model with cell-based tissue engineering of the bladder is needed in the near future. The third limitation is that only the ADEPCs were seeded onto the 3D scaffolds. Report showed that rapid anastomosis could be accomplished between the host vasculature and prevascularized networks on scaffolds co-cultured with endothelial cells and parenchyymmal cells[37]. Future study will consider the co-culture of ADEPCs and bladder parenchyymmal cells to facilitate the prevascularization process in vitro and the formation of vascular network between the scaffold and host bladder tissue in vivo. In addition, stem/progenitor cells will face autophagy, senescence, and reduction of stemness induced by the depletion of autocrine growth factors after culture for serial passage, it is necessary to perform karyotype analysis to the evaluate the possible structural and functional changes of ADEPCs after a long-term culture in vitro[38].

In summary, we have successfully cultured ADEPCs with high proliferative potential from the adipose tissue SVF. ADEPCs maintained the phenotypic and functional characteristic of EPCs and demonstrated proangiogenic potential in 3D scaffold. The results support the use of ADEPCs as angiogenic cell sources for engineering bladder tissue.

## Supporting Information

S1 Materials and Methods Detailed description of materials and methods for cell proliferation, immunofluorescence staining for cell phenotype, real-time quantitative reverse transcription PCR, and Matrigel-based capillary-like tube formation assay. (DOC)
(DOC)
