# Supplementary material for: In Vitro Evaluation of Endothelial Progenitor Cells from Adipose Tissue as Potential Angiogenic Cell Sources for Bladder Angiogenesis
Source: PLoS One. 2015 Feb 23;10(2):e0117644. doi: 10.1371/journal.pone.0117644 (PMC4338275; doi:10.1371/journal.pone.0117644)
Supplement: S1 Materials and Methods — (DOC) (DOC) [file pone.0117644.s001.doc]

**S1 Materials and methods**

Cell proliferation

ADEPCs, ADSCs and RBSMCs were seeded in 24-well plates at a density of 1250 cells/cm^2^ and cultured for 1, 2, 3, 4, 5, 6 and 7 days, respectively. The culture medium was changed every other day. At the indicated time, 10 μL of a Cell Counting Kit-8 (CCK-8; Dojindo Laboratories, Kumamoto, Japan) was added into each well (n=5), followed by incubation for 3 h. Then the incubated medium was transferred to a 96-well plate and the absorbance was measured at 450 nm with a reference wavelength at 620 nm (450/620 nm) using a Tecan Sunrise microplate reader (Tecan, Salzburg, Austria). Cell growth pattern, population doublings (PD), and doubling time (DT) were assayed. To calculate PD and DT, cell numbers and culture time were counted at passage 1 and 5. PD and DT were calculated as follows: PD=ln(N_f_/N_i_)/ln(2); DT=C_t_/PD. (N_f_: Final number of cells that were harvested, N_i_: Initial number of cells that were seeded, C_t_: Culture time that derives from the time interval between cell seeding and harvest)

Immunofluorescence staining for cell phenotype

ADEPCs, ADSCs and RBSMCs were cultured on glass slides in a 24-well plate pre-coated with 0.1% gelatin for facilitating cell adhesion. After the cells grew up to 50-60% confluence, they were fixed in 4% paraformaldehyde for 10 min, blocked and stained with primary membranous (CD31, CD34, Abcam) and cytoplasmic (stromal cell antigen [Stro-1], Millipore; endothelial nitric oxide synthase [eNOS], BD Biosciences; alpha-smooth muscle actin [α-SMA], Sigma-Aldrich) antibodies at 4℃ overnight. Cells were pretreated with 0.1% Triton-X100 for 5 min for permeability when cytoplasmic antibodies were used. Cells were then stained for 30 min at room temperature (RT) with secondary antibody conjugated with Alexa fluor 488 or Alexa fluor 633 (Both from Molecular Probes). Finally, 4’-6-diamidino-2-phenylindole (DAPI, Molecular Probes) staining was performed to detect the cellular nucleus. An IgG-matched isotype served as the internal control for each antibody.

Real-time quantitative reverse transcription PCR

Real-time quantitative reverse transcription PCR (RT-PCR) was performed to examine VEGF gene expression in ADEPCs, ADSCs and RBSMCs. Briefly, total RNA was extracted by using TRIzol reagent (Invitrogen) and converted into cDNA with Prime Script® RT Master Mix (TaKaRa) according to the manufacturer’s protocol. Then, a real-time RT-PCR reaction with final volume of 20 μl was completed by using the SYBR® *Premix Ex Taq*^TM^ (Tli RnaseH Plus) (TaKaRa). All reactions were performed in triplicate, and the thermal cycling conditions were 30s at 95℃, followed by 40 cycles of 5s at 95℃ and 34s at 60℃ using the Applied Biosystems 7500 real-time RT-PCR system.

Primer pairs were designed according to data from GenBank and evaluated by nucleotide BLAST standard search to avoid cross-reactivity with other known sequences. The designed sequences are as follows: VEGF (Forward: 5’-GGA GGA TGT CCT CAC TTG GA-3’, Reverse: 5’-CAG GCT CCT GAT TCT TCC AG-3’), β-actin (Forward: 5’-TGT CAC CAA CTG GGA CGA TA-3’, Reverse: 5’-TCT CAG CTG TGG TGG TGA AG-3’). For relative quantification, we compared the amount of target normalized to the β-actin amplification.

Matrigel-based capillary-like tube formation assay

To compare the different pro-angiogenic effects of ADEPCs, ADSCs and RBSMCs on endothelial cells, Matrigel-based capillary-like tube formation assay was performed using human umbilical vein endothelial cells (HUVECs). ADEPCs, ADSCs and RBSMCs were seeded in DMEM with 10% FBS at 1.5×10^5^ cells/well in 6-well culture plates. When 90% confluence was reached, the culture medium was removed and replaced with 2 ml of DMEM without FBS. After 24 hours culture, the medium was harvested and centrifuged at 1200 rpm for 10 min. The supernatant was collected and store at -80℃ until use (the storage dutation is less than one week). HUVECs were seeded quintuplicate onto solidified Matrigel at 6×10^4^ cells/well for Matrigel-based capillary-like tube formation assay. Cells were incubated with four different medium (DMEM without FBS (control group), ADEPCs CM, ADSCs CM and RBSMCs CM) for 10h to allow the formation of tubes. Three representative images were recorded from each well using phase-contrast.
